# PP10 Addressing The Challenge Of Environmental Sustainability Between Management And Health Technology Assessment At The Hospital Level

**DOI:** 10.1017/S0266462324001831

**Published:** 2025-01-07

**Authors:** Michela Bobini, Rossella Di Bidino, Iga Lipska, Americo Cicchetti

## Abstract

**Introduction:**

Several organizations specializing in health technology assessment (HTA), at various levels, are exploring avenues to integrate the dimension of environmental sustainability (ES) within their assessments. However, evidence remains scant on how best to do this. Across the different levels, hospital-based HTA (HB-HTA) also needs to comply with the leadership and strategies of the hospital and adapt to the existing resources and established partnerships.

**Methods:**

The purpose of this study is threefold: to provide an overview of the progress achieved by hospitals with respect to the integration of ES into HB-HTA; to detect consistencies between HB-HTA activities and hospital ES strategies; and to explore possible approaches and methods for integrating ES into HB-HTA. The methodology is structured in two steps: (i) a literature review on HB-HTA and environmental impact/sustainability to identify emerging approaches and methods for integrating environmental sustainability into HB-HTA; (ii) data collection by means of a questionnaire submitted to hospitals/organizations based on their membership of Health Technology Assessment International.

**Results:**

Preliminary results highlight a widespread awareness of the importance of integrating ES issues into HTA at the different levels at which it is performed. The incorporation of ES issues into HB-HTA is still in its infancy. There are some factors hindering the development of methods, such as the absence of scientific evidence on emerging approaches and the scarce availability of data or difficulty in tracing data back to a specific technology.

**Conclusions:**

The integration of the dimension of ES into HB-HTA still appears to be very immature. Coordination between the different actors in the system seems necessary to overcome the obstacles identified.

